# Deployment of Perioperative Nurses From Their Clinical Specialty During the COVID‐19 Pandemic: An Integrative Review

**DOI:** 10.1111/jan.17106

**Published:** 2025-06-09

**Authors:** Jennifer Crump, Debra Jackson, Maricris Algoso, Kath Peters

**Affiliations:** ^1^ Western Sydney University Sydney New South Wales Australia

**Keywords:** COVID‐19, deployment, disaster planning, experiences, integrative review, operating room nursing, pandemic, perceptions, perioperative nursing

## Abstract

**Aim:**

To identify the available records surrounding the deployment of perioperative nurses into differing clinical settings during the COVID‐19 pandemic.

**Design:**

Integrative review methodology.

**Methods:**

Quality appraisal of each record was conducted using a modified Critical Appraisal Skills Programme checklist. Data were extracted and presented based on outlined research objectives.

**Data Sources:**

Six electronic databases (CINAHL Plus, Google Scholar, MEDLINE, Pubmed, Scopus, and Web of Science) were searched, with relevant peer‐reviewed records published after 2019 until February 2025 included to differentiate from other respiratory pandemics.

**Results:**

Ten records were included in the review. Opposing discourse exists between perioperative decision makers and those perioperative nurses who underwent deployment to differing clinical areas surrounding perceptions and experiences of deployment during the COVID‐19 pandemic.

**Conclusion:**

Few studies exist exploring experiences of perioperative nurse deployment to a different clinical area during the COVID‐19 pandemic. Further research is vital to develop strategies that enhance the deployment process and ensure effective patient care across various clinical settings when cared for by deployed perioperative nurses.

**Implications for the Profession and/or Patient Care:**

Further research exploring transferable perioperative nursing skills and its subsequent influence on safe patient care may enhance and inform nurse deployment practices, enriching future staffing protocols in the event of a future pandemic.

**Reporting Method:**

PRISMA guidelines for reporting systematic reviews guided this review.

**Patient or Public Contribution:**

This study did not include patient or public involvement in its design, conduct, or reporting.

**Summary**

- Mixed perception exists regarding the deployment of perioperative nurses into differing clinical areas to care for patients during the COVID‐19 pandemic.
- This review highlights a global lack of high‐quality empirical research exploring the experience of perioperative nurse deployment.
- Further research about perioperative nurse deployment is imperative in understanding the impact that COVID‐19 related disruption had on the perioperative nursing workforce.

## 1 Introduction

COVID‐19 was declared a pandemic by the World Health Organisation (WHO) on March 11th, 2020, with the brief timeframe between localised and international spread causing significant strain, pressure, and disruption to health systems globally (World Health Organization 2023). Australia was well positioned to avoid the worst effects early within the pandemic, shielded by heavy border protection, suppression stratagems, low population density, and being an island nation (Fernandez et al. 2021; O'Sullivan et al. 2020). There was, however, an early impact of the pandemic on the Australian health system caused by two distinct peaks in 2020; the first occurred in March and April, seeing national emergence of the ancestral strain introduced via international travel, and the second from June to September, with community transmission impacting primarily the state of Victoria (Stobart and Duckett 2022). Suppression and elimination led to the eventual quash of the first wave of community transmission. The emergence of a highly transmissible Omicron subvariant in mid‐2021 increased the complexity of the national COVID‐19 elimination approach, with the COVID‐19 virus quarantine and circulating within urban populace settings of New South Wales and Victoria prior to completion of vaccination rollouts (Jefferies et al. 2023; Stobart and Duckett 2022).

COVID‐19 caused an influx of patients requiring urgent access to health care, with governing bodies required to rapidly implement strategic models of care to manage the increased demand for public health. Reallocation and deployment of the existing internal health workforce was identified by government and health leaders as the most pragmatic and efficient human‐resource approach (Jefferies et al. 2023; Kennedy et al. 2022). Within Australia, approximately 26,700 nurses work within perioperative services, with an estimated 14,000 perioperative nurses residing within New South Wales and Victoria, the two states that experienced the greatest need to deploy staff at the time of the Omicron community transmission (Australian Bureau of Statistics [ABS] 2021). Australian nurse registration requirements necessitate a generalist understanding across all areas of patient care, with many areas of nursing siloing into unique specialty practice areas. Perioperative nursing falls within the scope of critical care nursing, with nuanced sub‐specialty roles of anaesthetics, instrument, circulating, and post‐anaesthesia care requiring unique knowledge of care needs at distinctive points throughout a patient's surgical journey (Luk et al. 2025). Due to the complex nuances of each role, a nurse specialised in one distinctive perioperative role may not necessarily simply substitute another.

Due to the specialist nature of many nursing roles, transitioning nurses into alternate specialty areas would ideally require preparation, education and support; particularly where rapid deployment would require a nurse to be operational in clinical areas that historically require months‐to‐years of advanced professional training (Kennedy et al. 2022; Luk et al. 2025). Redeployment during the pandemic required perioperative nurses to not only convert from their nuanced perioperative specialist area, but also integrate into different clinical specialty areas outside of perioperative services, such as intensive care, who were experiencing pandemic overwhelm (Causby et al. 2024a; Kennedy et al. 2022). When blended with dynamic complexities of elective surgery closure, changes to workplace allocation, and evolving pandemic protocols, perioperative nurses underwent a distinctive ‘perfect storm’ of workplace impact.

While the COVID‐19 pandemic was deemed concluded by the Australian Chief Medical Officer on October 20, 2023, the impact of COVID‐19 on planned surgical waitlists remains omnipresent. Australian hospitals utilise the Activity Based Funding model, with hospitals relying on performing surgeries in a required timeframe to maintain each institution's financial health (NSW Agency for Clinical Innovation [ACI] 2024). Delays to planned procedures and surgeries impact both short‐and long‐term patient morbidity and mortality outcomes (NSW Agency for Clinical Innovation [ACI] 2024). Within Australia, 2022–2023 saw elective surgery wait times at their highest levels in over 20 years despite an 18% increase in elective surgery over the same period (Australian Institute of and Welfare 2023, 2024). These numbers highlight the essential need and critical operational role perioperative nurses play within the Australian health care system. It is vital to understand the impact of COVID‐19 workplace disruption and deployment on perioperative nurses as demand increases for nurses in this uniquely specialised area.

## 2 The Review

### 2.1 Aim

The aim of this integrative review was to identify the available evidence pertaining to workplace deployment of perioperative nurses into a new clinical area due to COVID‐19. The following questions further guided the review: (i) What were the perceptions and attitudes of decision makers surrounding perioperative nurse COVID‐19 deployment? (ii) Were there practice guidelines that underpinned deployment of perioperative nurses into new clinical areas(orientation, education, pre‐knowledge)? (iii) Were there reported implications for patient safety or quality when being cared for by perioperative nurses deployed outside of their usual skill set? (iv) What were the perceptions and attitudes of perioperative nurses who experienced COVID‐19 workplace deployment?

## 3 Methods/Methodology

### 3.1 Design

**FIGURE 1:**
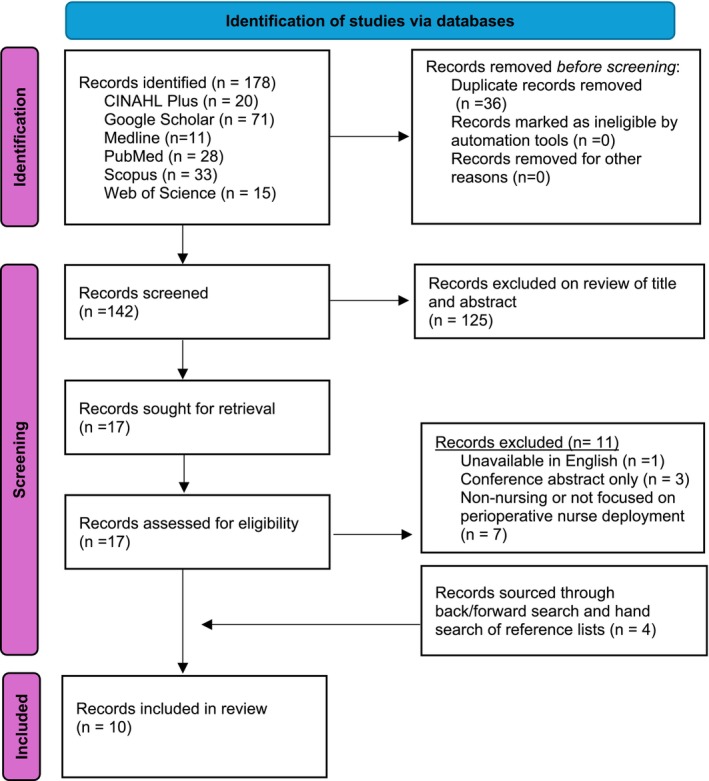
Preferred Reporting Items for Systematic Reviews and Meta‐Analyses (PRISMA) flow diagram.

An integrative review was selected as limited research focused on perioperative nurse deployment has been undertaken. Examining all original research was imperative to formulate a comprehensive understanding of the subject. The integrative review was underpinned by Whittemore and Knafl's 2005 framework (Whittemore and Knafl 2005). This framework outlined the five strategies undertaken for this review: outlining the problem, undertaking a search of literature, appraising data quality, data analysis, and synthesising presented findings. PRISMA guidelines for reporting systematic reviews guided this search (Figure 1).

### 3.2 Search Methods

The search was initially undertaken on August 20, 2024 with search term alerts capturing additional records until February 20, 2025. Search terms were developed in discussion with an academic librarian to ensure comprehensive capture of available literature. Terms used were nurse AND COVID‐19 OR SARS‐CoV‐2 OR Severe Acute Respiratory Syndrome AND disaster planning OR deployment OR secondment OR workplace assignment OR occupational disruption AND perioperative OR operating room OR operating theatre. Applicable synonyms, Boolean operators, wildcards, and truncations were applied to mitigate regional terminology variations. Papers were extracted from six databases: CINAHL Plus, Google Scholar, MEDLINE, Pubmed, Scopus, and Web of Science.

### 3.3 Inclusion/Exclusion Criteria

To be included in this review, records were required to address a minimum of one of the review's objectives; be focused on perioperative nurse deployment into a new clinical area resulting from the COVID‐19 pandemic; be published after 2019 to differentiate from other respiratory pandemics; have available full text; be peer‐reviewed; and be published in English. Qualitative, quantitative, mixed methods papers were eligible for inclusion. Case reports from peer‐reviewed sources were included due to the limited number of available records.

Excluded records were those that were not focused on perioperative nurse deployment; focused on other non‐nursing perioperative occupations; or focused on the deployment of nurses in a military context.

### 3.4 Search Outcome

The preliminary search located 178 papers from the six databases. Thirty six duplicates were removed, leaving 142 records for screening. Titles and abstracts of these records were assessed against the integrative review aims, with 125 eliminated. Seventeen records were retrieved for review. Of these, one record was unavailable in English, three records were conference abstracts only, and seven were non‐deployment or non‐nursing focused. Forward/backward searching of the retrieved records was undertaken, with hand searching sourcing four additional records meeting no less than one of the review's study aims. A total of ten records derived from Canada, Norway and North America were included. Of these, only three were studies suitable for critical appraisal.

### 3.5 Quality Appraisal

Records that met the review's inclusion criteria were read and reviewed to confirm eligibility. Each record underwent examination of merit using a modified Critical Appraisal Skills Program (CASP) tool adapted from Perez et al. (2019) (Table 1). The modified CASP tool improves visual data informatics and eliminates complexity when seeking assessment from both quantitative and qualitative reports (Critical Appraisal Skills Programme (CASP) 2023; Perez et al. 2019).

**TABLE 1:** Modified CASP appraisal tool.

Article	Clear aim/purpose	Methodology	Research design	Recruitment/sampling	Data collection	Reflexivity	Ethics	Data analysis, rigour/validity and reliability	Findings	Value of research	Overall quality
Clayton et al. (2022) (Canada)	*	N/A	N/A	N/A	N/A	N/A	N/A	N/A	N/A	*	Case study report
Danielsen and Vikan (2024) (Norway)	✓	✓	✓	✓	✓	*	*	*	✓	✓	B
Fiore‐Lopez (2021) (USA)	*	N/A	N/A	N/A	N/A	N/A	N/A	N/A	N/A	*	Case study report
Griffis et al. (2021) (USA)	✓	✓	*	*	*	X	X	*	*	✓	C
Hemingway and Silvestri (2021) (USA)	*	N/A	N/A	N/A	N/A	N/A	N/A	N/A	N/A	*	Case study report
Macasieb (2021) (USA)	*	N/A	N/A	N/A	N/A	N/A	N/A	N/A	N/A	*	Case study report
Peneza et al. (2021) (USA)	*	N/A	N/A	N/A	N/A	N/A	N/A	N/A	N/A	*	Case study report
Retzlaff (2020) (USA)	*	N/A	N/A	N/A	N/A	N/A	N/A	N/A	N/A	*	Case study report
Rollinson et al. (2021) (USA)	*	N/A	N/A	N/A	N/A	N/A	N/A	N/A	N/A	*	Case study report
Withall et al. (2023) (USA)	✓	✓	✓	✓	✓	✓	*	✓	✓	✓	A

*Note:* Checklist was adapted from Perez et al. (2019) based on the Critical Appraisal Skills Program (CASP) tool. *, screening question was covered but lacked detail; ✓, detailed coverage of screening question; A, nil or few flaws, the study credibility, transferability, dependability, and confirmability is high; B, some flaws, but unlikely to affect credibility, transferability, dependability and/or confirmability of the study; C, some flaws which may affect the credibility, transferability and/or confirmability of the study; D, significant flaws which are likely to affect credibility, transferability, dependability and/or confirmability of the study; X, screening question was not addressed.

### 3.6 Data Abstraction

Full text records were read and organised into a descriptive table listing the authors, publication date, the record's originating country, focus of the record, sample or participants (where relevant), and alignment with each question asked within the review's aims (Table 2).

**TABLE 2:** Abstraction and synthesis of retrieved records.

Author/year	Location	Method	Papers focus	Sample/participants	What were the perceptions and attitudes of decision makers surrounding perioperative nurse COVID‐19 deployment?	Were there practice guidelines that underpinned deployment of perioperative nurses into new clinical areas? (orientation/education/pre‐knowledge)	Were there reported implications for patient safety or quality when being cared for by perioperative nurses deployed outside of their usual skill set?	What were the perceptions/attitudes of perioperative nurses who experienced COVID‐19 workplace deployment?
1. Clayton et al. (2022)	Canada	Case study discussion.	1 hospital—North York General Hospital. 4‐month perioperative deployment in Alpha wave 2020. 3 months Jan‐March 2021.	—	+ Meeting involving senior executive and key perioperative stakeholders to notify/discuss deployment. + Senior executive wishing to display united front. + Utilise tiered team nursing model.	+ Perioperative educators performing independent skill assessment of each perioperative role—tailored response to identified needs. + Deployment pre‐education through self‐learning modules, in‐class learning, and simulation completed over 14 days pre‐deployment. + Spreadsheet to monitor/review skill sign off for deployed perioperative nurses. +Ward native nurses to provide support to deployment perioperative nurses.	+ Perioperative nurses to work under ‘team lead’ RN. Not to be independently responsible for patient care, rather holding ‘joint responsibility’. + Tasks were reassigned to senior nurse lead where perioperative nurse skill gap present to maintain quality patient care.	+ Not reported.
2. Danielsen and Vikan (2024)	Norway	Qualitative semi‐structured interviews.	To explore OR nurses experiences of their professional expertise utilisation while deployed in Wave 1 and 2. Data collected in May and June 2021. Length of deployment not noted.	13 perioperative nurses across 4 Norwegian Hospitals. Strategic sampling. All females. Ages 31–64. Perioperative experience < 1–30 years.	+ Not reported.	+ Not reported.	+ Reported little regard for patient safety or quality. + ‘Brief’ training then perioperative nurses left to manage ventilated patients alone. Does not state what the training was.	+ Prior clinical background in deployment area improved experience. + Those with less preparation/experience reported moral and workload stress. + PPE—deployed staff reporting shortages, management refuting this. + Medication safety challenges + Late‐stage role reallocation of perioperative nurses into roles where core competency of infection control could be utilised. + Mixed experience with communication/information flow and transparency. + Workload disparity—some deployments ‘heavy’ in ICU, others had ‘lighter’ computer‐based duties. + Healthcare personnel external to perioperative services unfamiliar with core competencies of operating room nurses.
3. Fiore‐Lopez (2021)	USA	Case study discussion	Discuss surge planning of St Charles Hospital in Long Island, NY, USA	—	+ Perceived PACU nurses would be primary pool for critical care deployment. + Attempt to uplift staff by playing specific songs when patients extubated and by assembling dance groups.	+ 10 days' notice to systematically cross‐train deployed perioperative staff. + PACU and other perioperative nurses with recent critical care experience sent to ICU. + Skill‐based practical training.	+ Not reported.	+ Not reported.
4. Griffis et al. (2021)	USA	Open ended survey question	To describe the experiences of RNs who were deployed out of their clinical specialty during COVID‐19. Deployment between 3 weeks and 6 months.	40 responses from deployed perioperative and procedural RNs.	+ Not reported.	+ Nil reported strategies—‘on the job’ training only.	+ Deployed nurses self‐reporting fear of adverse implications in absence of preparatory training and ongoing support.	+ Role strain in caring for patients outside of clinical specialty. + Unpreparedness exacerbating stress and psychological overload. + Fear at contracting virus. + Team bonding and collegiality.
5. Hemingway and Silvestri (2021)	USA	Case study discussion	Curriculum development for OR nurses to manage patient surges. 170 RNs requiring education in 2‐week period prior to deployment.	—	+ OR RNs deployed to labour pool – sent to call centre, respiratory clinic and in patient teams. + Rostering left to deployment ward. + Ward managers hosting deployed staff reported appreciation. + General care RNs were intended to be support staff only. + Care model changed, requiring OR nurses to take patient assignment.	+ Leaders identified OR nurses with recent (past 5 years) ICU experience to cover inpatient critical care. + Those without experience were deemed ‘general care RNs’ and allocated to specific unit for all deployment time. + ‘Experienced’ OR nurses remained in perioperative settings to cover critical operations. + 4–8‐h orientation sessions–minimum requirement for completion was online 4 h. + CNEs organised security access, lockers, scrubs, and hospital navigation/ward orientation for deployed OR staff. + Set up of peer‐support and regular emailing to deployed staff.	+ Liability protections enacted—health care professionals/facilities immune from civil liability regarding quality of care during COVID‐19 emergency. + Electronic documentation required additional support and source of stress.	+ Qualitative responses reporting overall positive experience despite unplanned patientassignment reallocation. Paper based on anecdotal feedback from nurse leaders.
6. Macasieb (2021)	USA	Editorial interview	Interview with 2 nurse leaders regarding how their facility deployed perioperative nurses into ICU. Macasieb & Duerson, March 2020.	—	+ Administrative leaders believed OR and PACU nurse skills would complement those of critical care and ICU, framing decision to deploy perioperative staff to ICU.	+Deployed perioperative nurses underwent classroom and hands on‐learning. Length of education not discussed. + ICU nurses required to provide support to deployed perioperative nurses.	+ States quality of care maintained, and perioperative nurses were successful in their deployment.	+ Anecdotal report of deployment benefits regarding gained skill and knowledge.
7. Peneza et al. (2021)	USA	Case study discussion	Information on perioperative nurse educator roles and responses during the pandemic.	—	+ Chief Nursing Officer directed deployed perioperative nurses to non‐patient care infection control roles for inpatient areas.	+ Nurse educators and hospital leadership identified appropriate locations where OR staff could provide care aligned with skillset. + General organisational orientation reduced from 4 days to 2 days for rapid staff onboarding. + Acknowledged plan was required for deployment—cannot reallocate from OR without knowledge and skill, however deployed OR staff to ED.	+ Not reported.	+ Not reported.
8. Retzlaff (2020)	USA	Special report	Hospital leaders' descriptions of morale boosting activities they undertook for deployed OR staff		+ Director of Surgery reported PACU nurses had ‘easy’ transition as had ICU background. + Psychological care to deployed OR staff: (i) allowing access to more PPE than minimum requirements (wearing of gowns and cloth hats). (ii) leaders standing at door holding hero signs. (iii) food rewards and showbags. (iv) wellness area with soft music. + Management noting deployed staff can't wait to get back to doing their pre‐deployment work that they love to do.	+ Utilisation of ‘nurse partnership model’ to pair deployed OR nurse with ICU nurse. + All deployed PACU nurses had ICU background. + Deployed OR staff self‐completing orientation checklists	+ Not reported	+ Not reported
9. Rollinson et al. (2021)	USA	Case discussion of John Hopkins Hospital	Discussion of redeployment of 113 CRNAs into RT and ICU RN roles during pandemic.	—	+ Deployment of CRNAs was seen as strategy to prevent involuntary furlough of staff.	+ 10‐week ICU orientation condensed to 2 to 3 days. + CRNAs staffed with experienced RT for even workload team‐based care. + ICU deployed CRNAs paired with non‐ICU nurse or had own patient assignment.	+ CRNAs reported detecting patient deterioration missed by non‐critical care trained staff.	+ Survey of CRNAs reported ‘more than half’ of CRNAs were willing to be deployed. + Physical discomfort from PPE (dry eyes and headaches). + Deployed CRNAs enjoyed educating others about professional role of a CRNA, inspiring other RNs to pursue this career path.
10. Withall et al. (2023)	USA	Qualitative phenomenological design using Colizzi's method. Semi‐structured interviews	Describe how perioperative and ambulatory care RN experiences of caring for COVID‐19 patients in acute care settings in 2020	6 perioperative nurses. 2 ambulatory care nurses. Researchers contacted potential participants. Snowball sampling. 6 female and 2 males in study	+ Nil reported (Deployed nurse leaders excluded from study).	+ Compressed 2‐day orientation when deployed. + Initial care model had perioperative as care extenders only. Changed to team‐based model with shared patient assignment.	+ Nurses experienced moral distress as concerned patients receiving suboptimal care. + OR nurse reported they did not have competency or skills required to care for patients on inpatient units. + Reported OR nurse skill advantage of sterile technique which benefited patients.	+ Preparatory orientation seen as inadequate. + Disruption to usual work routines at short notice. + Trauma at seeing colleagues' contract and die of COVID‐19. + Self‐care and sense of accomplishment regarding their resilience on deployment.
Total	Canada *n* = 1. Norway *n* = 1. USA *n* = 8.	Case papers *n* = 7. Research papers *n* = 3			7	8	7	6

Abbreviations: CNE‐ Clinical Nurse Educator; CRNA—Certified Registered Nurse Anaesthetist; ICU—Intensive Care Unit; OR—Operating Room; PACU—Post Anaesthesia Care Unit (Recovery); PPE‐ Personal Protective Equipment; RN—Registered Nurse; RT‐ Respiratory Therapist.

## 4 Results/Findings

### 4.1 Perceptions and Attitudes of Decision Makers

Seven papers described insights from perioperative services decision makers about deploying perioperative nurses. All papers were sourced from North America and were case reports or brief interviews. Decision makers in these papers were hospital nursing executives, surgical directors, perioperative nurse managers and nurse educators. Decision makers considered perioperative nurses as best skilled to mitigate staffing deficits (Clayton et al. 2022; Fiore‐Lopez 2021; Macasieb 2021; Retzlaff 2020; Rollinson et al. 2021). Surgical directors believed nurses with prior experience in Post Anaesthesia Care Units (PACU) or nurses with prior critical care experience would have the easiest transition into COVID‐19—impacted critical care environments (Retzlaff 2020). Further, deployment was utilised to prevent permanent involuntary lay‐off of skilled nurses during elective surgery downtime (Rollinson et al. 2021). Two papers anticipated perioperative nurses would experience deployment transition difficulties due to their uniquely specialised skillset. Decision makers in these papers believed allocating deployed nurses specifically into task‐focused non‐patient care areas, such as infection control roles or protocol‐led clinic positions, would best utilise perioperative nursing skills without compromising on patient safety (Hemingway and Silvestri 2021; Peneza et al. 2021). Overt displays of support by decision makers towards deployed staff were enacted as a source of staff motivation. These included food rewards, inspirational signage, staff wellness areas, and organised dancing that were enacted to inspire those who were working in pandemic‐impacted work areas (Clayton et al. 2022; Retzlaff 2020) Concerningly, one paper outlined how decision makers utilised Personal Protective Equipment (PPE) as a tool of gratuity, with provision of PPE above the minimum safety requirement used as a demonstration of goodwill to deployed staff (Retzlaff 2020).

### 4.2 Practice Guidelines

Eight papers discussed practice guidelines that would underpin perioperative nurse deployment. Systematic compressed upskilling of perioperative nurses was reported in each of these papers; and ranged from self‐directed learning (Clayton et al. 2022) to onsite skill based training (Fiore‐Lopez 2021). Advanced Practice Certified Registered Nurse Anaesthetists (CRNAs) were the only deployed cohort requiring minor additional pre‐deployment training (Rollinson et al. 2021). Clinical Nurse Educators bore the weight of rapid deployment education, and attempted to implement critical care supplemental training, which usually took months, in as little as two days (Rollinson et al. 2021). There were instances of no pre‐emptive training, where nurses learned of their reallocation at the time of deployment into a new clinical area (Griffis et al. 2021). Where training was implemented, upskilling education was often intended only to upskill deployed nurses into supportive care‐extender roles, rather than autonomous critical care practitioners (Hemingway and Silvestri 2021; Macasieb 2021; Retzlaff 2020; Withall et al. 2023). Staff ‘experienced’ in operating theatres remained within perioperative services to manage emergent case theatre workload (Hemingway and Silvestri 2021).

There were reports of no orientation and suboptimal assimilation into new clinical areas for some deployed perioperative nurses (Griffis et al. 2021; Withall et al. 2023). For others, there was use of new staffing models where deployed perioperative nurses would fall under a shared patient care approach with a nurse ‘native’ to the area. One single site Canadian report paper utilised a team nursing model, whereby an experienced nurse 'native' to the receiving clinical area coordinated care with deployed staff in a mutually dependent capacity (Clayton et al. 2022). Deployed perioperative staff were expected to hold joint responsibility within this care model. In instances where perioperative nurse upskilling into shared care was time inefficient or deemed unsafe, they were to be immediately reallocated into a task‐focused supplementary role under the direct supervision of a ward's ‘native’ senior nurse (Clayton et al. 2022). Likewise, an American‐based care model saw nurses graded into three different roles: support nurse, nurse partner, or a unit‐based nurse ‘native’ to the unit (Hemingway and Silvestri 2021). Perioperative nurses became nurse partners within this model, required to hold shared responsibility and collaborative decision with the unit‐based ‘native’ nurse. This arrangement was seen as essential surrounding tasks perioperative nurses were not usually accustomed to, such as medication administration and ventilation care. Some deployed perioperative nurses were allocated into a support‐only nurse role, operating in an auxiliary capacity without a patient load. Support nurses involved roles previously identified as aligned with perioperative skills, such as infection control monitors, equipment officers, and supporting patient tasks associated with activities of daily living (Peneza et al. 2021). Each deployed nurse received a rapid orientation to their new clinical area that included security access and ward logistic housekeeping and were followed up with peer support to maintain connection to their home‐unit colleagues and leaders (Hemingway and Silvestri 2021).

### 4.3 Patient Safety

Seven papers retrieved commented on patient safety when cared for by deployed perioperative nurses, with varying accounts of welfare presented. Within the USA, the enactment of emergency liability protections in several states afforded deployed clinicians legal safeguards against civil lawsuits (Hemingway and Silvestri 2021; Macasieb 2021). Case reports from the viewpoint of decision makers described an acceptable level of care from deployed perioperative nurses in the context of the emergency circumstances (Hemingway and Silvestri 2021; Macasieb 2021). Decision makers affirmed patient reassignment would occur should a care task or patient acuity surpass the expertise of the deployed perioperative nurse as part of shared‐care staff models (Clayton et al. 2022).

Concern for undetected patient deterioration was discussed in three qualitative papers, where actual or potential adverse outcomes were reported by deployed perioperative nurses working outside of their usual clinical expertise (Danielsen and Vikan 2024; Griffis et al. 2021; Withall et al. 2023). Norwegian deployed perioperative nurses stated clinical decision makers had little regard for patient safety or quality, and reported being left to independently manage ventilated patients without prior training or supervision to safely support the provision of this care (Danielsen and Vikan 2024). Withall et al. (2023) explicitly affirmed for their North American nurses that without considerable upskill training and supportive supervision, perioperative nurses working outside of their usual clinical expertise do not have the required skill or competency to provide independent care for inpatients (Withall et al. 2023). Advanced practice CRNAs holding specialty training in critical care reported averting patient deterioration near‐miss events undetected by deployed perioperative staff inexpert in critical care (Rollinson et al. 2021).

### 4.4 Perceptions and Attitudes of Deployed Perioperative Nurses

Six papers were retrieved that discussed perceptions and attitudes of perioperative nurses related to deployment. Of these, two case papers authored by perioperative decision makers reported overall positive feedback from deployed nurses. Hemingway and Silvestri (2021) quoted anecdotal feedback received from their deployed perioperative staff feeling appreciated and receiving kind treatment in their deployment area (Hemingway and Silvestri 2021). Likewise, Retzlaff (2020) reported deployment as a beneficial avenue for professional development, new knowledge, and skill enhancement opportunities (Retzlaff 2020). The remaining four papers reported a differing perception of events. Where reallocation preparation was not provided, perioperative nurses reported a sense of chaos; however, they did acknowledge professional support capacity was limited in the emergent pandemic circumstances (Griffis et al. 2021). Prior clinical experience and skillset aligned to the deployment area, such as prior ward or critical care experience, was a protective factor for deployed perioperative nurses (Danielsen and Vikan 2024; Griffis et al. 2021; Rollinson et al. 2021). CRNAs were the only perioperative nurse cohort to report confident practice in critical care areas despite initial deployment apprehension. CRNAs utilised deployment as an opportunity to promote their niche specialty skills to peers in other areas (Rollinson et al. 2021). Perioperative nurses with limited nursing experience outside of perioperative areas suffered emotional distress and moral injury due to their limitations in knowledge, particularly surrounding care of patients in critical care areas and medication management (Danielsen and Vikan 2024; Griffis et al. 2021; Withall et al. 2023).

Contracting COVID‐19 on deployment was an underpinning fear for reallocated nurses and made protective equipment accessibility a prime concern. Physical pain from using protective equipment, such as headaches and ocular discomfort, was reported but was an accepted working condition within a pandemic (Rollinson et al. 2021). Some deployed nurses reported inadequate supplies and being denied access to protective equipment despite clinical leaders refuting that no such issue existed (Danielsen and Vikan 2024). Fear was compounded by witnessing colleagues contract, become ill, and die from COVID‐19, amplifying the dangerous reality of occupational exposure with limited PPE provision (Withall et al. 2023).

## 5 Discussion

The aim of this review was to examine available peer‐reviewed records and critically synthesise available research literature pertaining to perioperative nurse deployment outside of their perioperative service clinical specialty due to COVID‐19. No other literature review focused on COVID‐19 pandemic deployment of the perioperative nursing workforce was located while conducting this review, highlighting a global dearth of high‐quality research exploring this subject.

Deployment experiences reported by perioperative decision makers and those perioperative nurses who underwent deployment highlight a dissonance of opinion. Perioperative decision makers believed PACU and CRNAs were best placed for an uncomplicated transition into critical care areas founded on an assumed pre‐existing skillset (Fiore‐Lopez 2021). Autonomous CRNA roles are predominantly local to North America, and while similar roles exist in other countries, their education and clinical scope vary (Michaels and Foran 2022). Anaesthetic nurses within the Australian context often interchange roles with PACU nurses and range in educational background and training as Endorsed Enrolled Nurses (Diploma of Nursing), Registered Nurses (Bachelor of Nursing) and Nurse Practitioners (Masters of Nursing). Australian anaesthetic and PACU nurses are not generally required to hold additional specialist nurse qualifications and need only engage in 20 h of continued professional development activities to maintain their position (Michaels and Foran 2022). Due to local variances in training, role descriptions, and practice scope, Australian perioperative decision makers simply cannot benchmark against international literature to determine which perioperative specialty can be seamlessly deployed to alternate critical care areas. There was no specific reference to deployment perceptions of instrument or circulating nurse roles located as part of this review. There is a dearth of literature guiding the scope of practice for deployed perioperative nurses into new clinical areas, which requires further exploration.

Personal Protective Equipment (PPE) access was a contentious issue throughout the pandemic, with deployed perioperative nurses voicing concern at poor provision and access to basic protective equipment (Danielsen and Vikan 2024). Deployed perioperative nurses reported inadequate access to PPE and denial from decision makers that shortages in safety equipment existed (Danielsen and Vikan 2024; Withall et al. 2023). Perioperative nurses hold intrinsic knowledge at the importance infection control, with PPE use a core component daily clinical practice within perioperative areas. A known global PPE shortages and disruption to the supply chain occurred during the pandemic which supports deployed perioperative nurses PPE shortage claims (Cohen and Rodgers 2020; Withall et al. 2023). Shortages saw North American nurses protesting due to insufficient provision of PPE, likening the shortage to soldiers fighting in wartime combat with cardboard body armour (Cohen and Rodgers 2020). PPE shortages saw nurses seek PPE from alternate sources such as hardware stores, where the majority of available supplies are designed for the Caucasian male physique (Cohen and Rodgers 2020). As a female dominated and culturally diverse profession, access to correctly fitted PPE in a pandemic holds racial and gendered impact for nurses. Government health and safety policy within Australia highlight reasonable expectation for workers presuming provision of comfortable and correctly fitted PPE for hazardous occupational exposure ‘so far as reasonably practicable’ (Clinical Excellence Commission (CEC) 2024). While Australian clinical governance neither recommend nor endorsed reuse of single usage protective items, they acknowledge crisis shortages of equipment may require change usual PPE practice and provision for health care workers (Clinical Excellence Commission (CEC) 2024).

Perceptions of adequate patient safety received conflicting responses within the retrieved literature. Emergency adjustment to medico‐legal liability during the pandemic indicated understanding of suboptimal patient care during this period (Hemingway and Silvestri 2021). However, papers reporting the views of perioperative decision makers believed patients would have access to an adequately trained practitioner, safety netted by a reassignment of patient care when clinical acuity exceeded the skill capability of a deployed nurse. Higher quality papers sourced in this review reported assumption surrounding this model of care was erroneous, with deployed perioperative nurses detailing instances of independently managing ventilated patients without perceived supervision, support, or the technical aptitude to do so. Scope of practice ambiguity implied deployed nurses were not to practice outside of their clinical knowledge and that critical care nurses would feasibly be able to train deployed nurses. Participants in an Australian study by Causby et al. (2024a) reported intense pressure for the ‘native’ unit nursing team to hold dual responsibility in simultaneously supporting deployed nurses and caring for critical patients, reporting ambiguity surrounding scope of practice and accountability surrounding patient care having a significant toll on the critical care workforce (Causby et al. 2024a). Simplistic models of task allocation hold no guarantee of success when care is allocated to individuals unfamiliar with a task's complexity, such as was experienced by perioperative nurses unfamiliar with the unit or patient care needs.

Literature included in this review highlight both perioperative decision makers and nurses recognised the necessity of preparatory training to successfully integrate deployed nurses into a new clinical area, however there were varying degrees of preparatory training success. The surge of COVID‐19 patients across Asia, Europe, the Americas and Africa justifiably challenged hospitals to provide required training in time. As an island nation, Australia was one of the few countries in a position to pursue an eliminatory ‘zero‐COVID’ approach, with seven COVID‐19 deaths recorded in Australia between December 2020 to June 2021 compared to the international estimate of 3.4 million deaths over the same period (Australian Bureau of Statistics [ABS] 2021; World Health Organization 2023). This respite interval offered Australian health agencies opportunity to prepare and upskill their nursing staff for probable work reallocation as the nation transitioned from an elimination strategy to a suppression approach. Despite preparatory opportunity, Australian nurses who experienced deployment from medical/surgical areas describing an unsatisfactory deployment experience influenced by perceived inadequate orientation and preparatory training to care for unfamiliar patient complexities (Chu et al. 2023). Causby et al. (2024b) affirm this, highlighting few studies exist exploring preparedness of non‐critical care nurses deployed to critical care areas during the pandemic (Causby et al. 2024b). The question remains what reskill training was received by Australian perioperative nurses on deployment in differing clinical areas, and where provided it's perceived efficacy in supporting perioperative nurses to provide safe patient care in their deployment area.

### 5.1 Implications for Practice

The current literature draws attention to the role deployment of nurses played in staffing hospitals across the world during the COVID‐19 pandemic. This review brings to light inconsistent discourse surrounding the deployment of perioperative nurses into new clinical areas during the COVID‐19 pandemic. Limited information exists exploring the experiences of perioperative nurses who underwent deployment into new clinical areas during the pandemic, how they utilised their perioperative skills on deployment, and their perception of providing patient care in settings outside of perioperative areas. Nurses are likely to be called upon in future pandemic situations to rapidly adapt to new clinical roles outside of their usual clinical speciality, with understanding how to best utilise the perioperative nursing workforce guiding future training and policy development. Further research exploring preparatory training, transferable perioperative nursing skills between acute and critical care settings and its subsequent influence on safe patient care may enhance future perioperative nurse deployment during staffing shortfalls, inform cross‐training opportunities for perioperative nurses, and enhance future safety protocols to support surge patient needs.

### 5.2 Limitations

This review presents some limitations that may impact on results. The authors only considered peer‐reviewed records available in English; however, only one article was excluded on this basis. Nine of the ten records that met the study aims originated from North America, limiting the understanding of perioperative nurse experiences in the context of a global pandemic. While studies were appraised with the CASP tool, most retrieved records were unsuitable to be evaluated due to a lack of qualitative, quantitative, or mixed methods research exploring perioperative nurse deployment during the COVID‐19 pandemic.

## 6 Conclusion

The deployment of nurses was prevalent worldwide as a staffing strategy to manage the influx of patients presenting to health care facilities during the COVID‐19 pandemic, with planned surgery reduction allowing perioperative nurses to be reallocated in supporting staffing shortfalls. Inconsistent accounts exist between perioperative decision makers and nurses who experienced deployment surrounding preparatory training, occupational safety and patient care. Overall, little is reported about the experiences of perioperative nurses who were deployed into new clinical areas during the COVID‐19 pandemic, the transferability of their perioperative skillset in differing clinical areas, or their ability to safely care for patients with care requirements differing from what these nurses usually experience within operating rooms. Further research is vital to develop strategies that enhance the deployment process and ensure effective patient care across various clinical settings when cared for by a deployed perioperative nurse.

## Author Contributions

Made substantial contributions to conception and design, or acquisition of data, or analysis and interpretation of data: J.C., D.J., M.A. and K.P.; Involved in drafting the manuscript or revising it critically for important intellectual content: J.C., D.J., M.A. and K.P.; Given final approval of the version to be published. Each author should have participated sufficiently in the work to take public responsibility for appropriate portions of the content: J.C., D.J., M.A. and K.P.; Agreed to be accountable for all aspects of the work in ensuring that questions related to the accuracy or integrity of any part of the work are appropriately investigated and resolved: J.C., D.J., M.A. and K.P.

## Conflicts of Interest

The authors declare no conflicts of interest.

## Data Availability

The data that support the findings of this study are available on request from the corresponding author. The data are not publicly available due to privacy or ethical restrictions.
